# Spatial hearing adaptation in congenital and acquired single-sided deafness

**DOI:** 10.3389/fnins.2026.1726012

**Published:** 2026-02-27

**Authors:** Sebastian A. Ausili, Sandra M. Prentiss, John J. Sheets, Hillary A. Snapp

**Affiliations:** Department of Otolaryngology, University of Miami Health System, Miami, FL, United States

**Keywords:** cochlear implant, congenital deafness, monaural cues, single-sided deafness, spatial hearing, sound localization

## Abstract

**Introduction:**

Spatial hearing depends on binaural integration of interaural time and level differences and monaural spectral cues. Single-sided deafness (SSD) disrupts this process, impairing localization and speech perception. Congenital (SSDc) and acquired (SSDa) SSD provide unique models for studying cortical adaptation, while cochlear implantation (CI) offers partial restoration of binaural hearing. This study examined differences in localization performance and response promptness across SSDc, SSDa, and SSD-CI listeners.

**Methods:**

Thirty-one SSD listeners (9 SSDc, 11 SSDa, and 11 SSD-CI) and 16 normal-hearing (NH) controls completed broadband noise localization tasks in azimuth (90°) and elevation (30°). Azimuth and elevation localization, bias, mean absolute error (MAE), and response promptness were analyzed using linear regression and mixed-effects models.

**Results:**

SSD-CI listeners with CI on demonstrated significantly improved azimuth localization compared to CI off (gain = 0.97 *vs.* 0.26, MAE = 27°, *vs.* 47°, *p* < 0.001). SSDc listeners performed more accurately than SSDa (gain = 0.57 *vs.* 0.17, *p* < 0.001), consistent with enhanced adaptation to monaural cues following early deprivation. All SSD groups showed poor vertical localization on the deaf side (gain = 0.15) and slower responses (mean = 2.17 s^−1^), compared to NH (4.02 s^−1^, *p* < 0.037). CI was associated with faster responses, suggesting improved processing efficiency.

**Discussion:**

Early deprivation in SSDc promotes compensatory strategies for spatial hearing, while SSDa shows more limited adaptation. CI can partially reintroduce binaural cues, improving horizontal localization and processing efficiency, though vertical localization deficits persist.

**Conclusion:**

These findings demonstrate that auditory experience and hearing restoration jointly influence spatial hearing behavior. Understanding these adaptive patterns can guide individualized rehabilitation strategies and optimize outcomes for individuals with unilateral hearing loss.

## 1 Introduction

Spatial hearing, the ability to localize sound in the three-dimensional space, is a critical auditory function that relies on the integration of interaural time differences (ITDs) and interaural level differences (ILDs), along with, monaural spectral-shape cues arising from the high-frequency (>4 kHz) sound wave interactions with the head and pinnae (Blauert, 1996a; Blauert, 1996b). In individuals with normal hearing (NH), these cues combine to enable precise sound localization in both the horizontal (azimuth) and vertical (elevation) planes. However, unilateral sensorineural hearing loss, or single-sided deafness (SSD) disrupts this process, leading to significant challenges in sound localization (Snapp and Ausili, 2020) and speech understanding in noisy environments (Wie et al., 2010; Rothpletz et al., 2012; Reeder et al., 2015; Alhanbali et al., 2017). Without binaural hearing, SSD listeners are restricted to monaural spectral cues, which are less effective for accurate localization, particularly in complex auditory environments (Van Wanrooij and Opstal, 2004). SSD manifests in two forms: congenital (SSDc) or acquired (SSDa), which can be treated with interventions like cochlear implants (SSD-CI). These conditions provide a unique framework to study cortical plasticity and compensatory mechanisms that enable adaptation to altered auditory inputs.

Present from birth, SSDc occurs during critical periods of auditory development, allowing the brain to reorganize and potentially enhance the use of monaural cues for sound localization (Kral et al., 2013a; Kral et al., 2013b). In contrast, when SSD is acquired after auditory system maturation, the window for cortical remapping is limited due to reduced plasticity, presumably leading to poorer localization performance (Keating and King, 2013). Prior work in children with early onset SSD demonstrates an “aural preference” reorganization toward the hearing ear, with weaker central representation of the deaf ear (Gordon et al., 2015). Notably, early compensation in SSDc may reduce the perceived auditory handicap, potentially diminishing the relative benefit of interventions like cochlear implantation later in life. For instance, individuals with SSDc who develop robust monaural cue utilization may experience less functional impairment, making the additional binaural input from a CI less transformative compared to those with SSDa.

Cochlear implantation (CI) has emerged as a restorative approach by partially reintroducing binaural input, enabling the integration of ILDs (Arndt et al., 2017; Snapp and Ausili, 2020). However, while CIs significantly improve localization compared to the CI-off condition, they only partially restore binaural hearing due to limitations in spectral resolution and timing (Francart et al., 2018; Gifford and Stecker, 2020). The degree of benefit from CIs may vary based on SSD etiology and the extent of pre-existing compensatory mechanisms, raising critical questions about the optimal candidacy for implantation.

Most localization studies in SSD listeners have focused on post-lingually deafened adults (Oh et al., 2023; Daher et al., 2023), while the spatial hearing capabilities in azimuth and elevation of SSDc individuals are rarely examined. SSDc may develop compensatory mechanisms over time, such as enhanced sensitivity to head-shadow or spectral cues, due to early exposure to unilateral hearing. In contrast, SSDa listeners who experience hearing loss after years of binaural hearing may struggle to develop these adaptations, leading to persistent handicap.

In this study, we investigate sound localization performance in SSDc, SSDa, and SSD-CI listeners. We hypothesize that SSDc listeners will exhibit stronger compensatory mechanisms due to early cortical adaptation, evidenced by better target-response relationship and lower response variability compared to SSDa. By examining differences in localization performance and response promptness across SSD profiles and CI intervention, this study aims to elucidate the interplay of cortical reorganization, compensatory mechanisms and CI efficacy.

## 2 Methods

### 2.1 Participants

A total of 31 single-sided deaf (SSD) listeners participated in the study (16 males and 15 females; M = 36.3 years, SD = 15.8 years). All participants were less than 65 years of age and had normal audiometric thresholds (< 20 dB HL) from 0.125 kHz to 8 kHz in the better hearing ear and severe to profound hearing loss in the impaired ear. There were 9 congenitally (i.e., at birth) deafened listeners (SSDc) and 22 with adult-onset SSD. Of those with acquired SSD, 11 were untreated (SSDa), and 11 were treated with a CI (SSD-CI). All CI users had at least 6 months of device experience, and all listeners had at least 12 months of SSD listening experience. A control group of bilaterally NH listeners (7 males and 9 females; ages 22–42 years) with pure tone audiometric thresholds < 20 dB 0.125 kHz—8 kHz bilaterally were also included. None of the SSD listeners or NH controls had visual or motor disorders and all were naïve about the purpose of the experiments.

### 2.2 Experimental set-up

**Figure 1:**
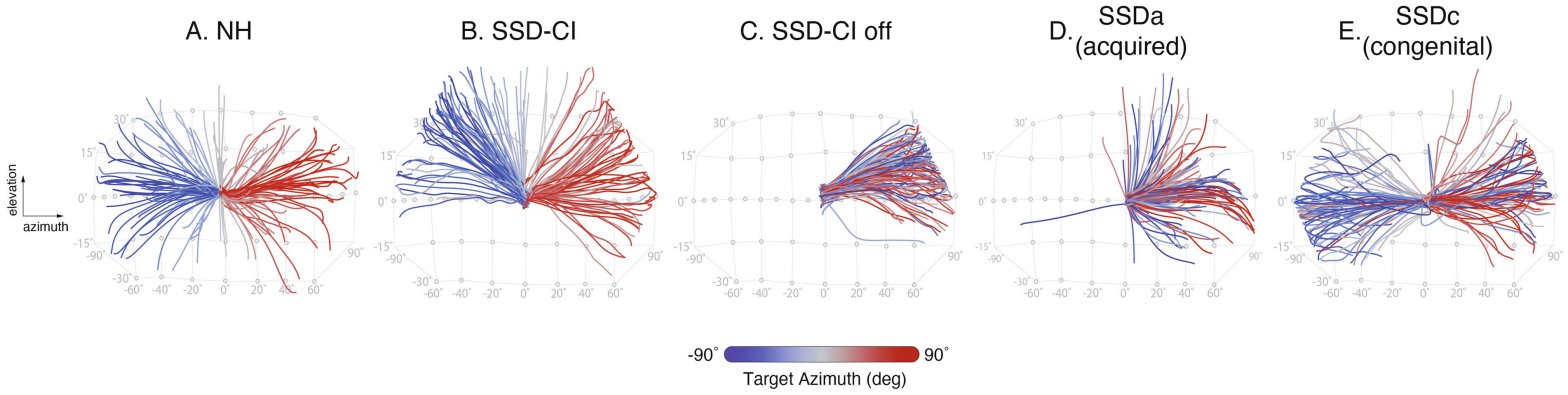
Representative listener examples are shown for the experimental groups. **(A)** NH (normal hearing), **(B)** SSD-CI, **(C)** SSD-CI off, **(D)** SSDa (acquired), and **(E)** SSDc (congenital). The grid and grayscale circles depict the experimental set-up: 46 loudspeakers arranged in an array spanning –90° to +90° azimuth (10° minimum separation) and –30° to +30° elevation (15° minimum separation) relative to the listener’s head. Each circle marks a specific speaker location. Colored lines illustrate representative head orientation trajectories recorded from participants in response to leftward (blue) and rightward (red) auditory targets. Head orientation is referenced to the actual response location, enabling visualization of response strategies across listening groups.

The experiments took place in a sound-attenuated auditory booth (4.3 × 4.3 × 2 m) with a spherical array of 72 speakers (e301, KEF, Maidstone, England) of 1.3 m in radius. For the purposes of this experiment, 46 speakers spanning ±90° in azimuth and ±30° in elevation were used to present sound stimuli (Figure 1). Audio signals were driven by a Focusrite RedNet PCIeR (Focusrite, High Wycombe, England) and Sonible d: 24 multi-channel amplifiers (Sonible, Graz, Austria) at as 44.1 kHz sampling rate. Stimuli presentation was implemented in MATLAB (ver. R2022a, The MathWorks, Natick, USA) using the Psychtoolbox extension (Brainard, 1997; Pelli, 1997; Kleiner et al., 2007).

Head movements were recorded using a Flex 3 motion tracking system (Optitrack, Natural Point Inc., Corvallis, OR, USA) with six cameras positioned around the subject, providing 360° coverage to allow precise tracking of head position and orientation in six degrees of freedom. Data were acquired at a sampling rate of 100 Hz. Participants wore a headband fitted with small retroreflective markers defining a rigid body, allowing an estimation of head position with a three-dimensional accuracy of +/−0.50 mm. A small laser pointer mounted on the head band provided visual feedback to facilitate alignment of gaze with head orientation during task. The tracking system was synchronized with stimulus presentation using the Lab Streaming Layer (Kothe et al., 2025).

#### 2.2.1 Stimuli

Stimuli consisted of 150 ms broadband gaussian white noise bursts (BB, 0.2 to 20 kHz) presented at 50 dBA, 60 dBA, and 70 dBA for each of the 46 speaker locations, totaling 138 trials per condition (46 locations × 3 intensities). Each speaker was positioned at a unique combination of azimuth and elevation relative to the listener’s head; notably, azimuth speakers spanned −90° to 90° with 10° separation while elevation ranged −30° to 30° separated by 15° (Figure 1, grey circles). Sound pressure levels were measured using an SPL meter (NSRTW mk4, Convergence Instrument, Sherbrooke, Canada) held at the center position of the speaker array.

#### 2.2.2 Sound localization paradigm

Listeners were positioned in the center of the speaker array in a dark sound-attenuated room to remove response bias and eliminate visual cues during the experiment. The headband’s laser was used for central fixation and to indicate the perceived location of the auditory stimuli. Each experimental session was calibrated for the individual listener head positioning to the target locations. At the start of each trial, listeners were instructed to fixate the laser on the head tracker to the central (baseline) target presented straight ahead at 0° azimuth, 0° elevation. Stimuli were interleaved and presented randomly from the 46 speakers. Listeners self-advanced the trials using a handheld trigger and were instructed to orient their head as fast and as accurately as possible to the perceived sound location. The acquisition time for head movements was 1.5 s, after which the central fixation LED was switched on to reorient the listener to the baseline central position before advancing to the next trial. The onset of each sound stimulus was delayed by a randomized interval (200–300 ms) to control for predictive effects on response times.

### 2.3 Data analysis

Most of the data analysis procedures employed in this study have been previously described in detail in Ausili et al. (2019) and are briefly summarized below. To assess sound localization stimulus–response relationship in azimuth, a linear regression was performed using the least-squared error criterion on the location data points (Equation 1). The regression was performed on the target angle (*α*T) and the response angle (αR) as follows:


$$\alpha_{R}=b+g\times \alpha_{T}$$ (1)

Here, g represents the slope or gain of the best-fit regression line (dimensionless), quantifying the degree to which participants’ responses track changes in target location; a gain of 1 indicates accurate localization responses, while a gain near 0 reflects little correspondence between target and response. Bias (b) represents the intercept (in degrees) and reflects a systematic spatial shift in responses, such as a consistent deviation toward one side; a bias of 0° indicates no systematic shift. A perfect localization response would result in a gain of *g* = 1 and a bias of *b* = 0°.

To evaluate whether elevation localization varied with azimuth, elevation responses were analyzed by fitting linear regressions across the full elevation range (−30° < *ε* < 30°) within nine contiguous, 15° wide azimuth windows spanning the frontal hemifield (−60° to +60°). These windows provided localized estimates of elevation performance while preserving sufficient data within each azimuthal region for regression fits. This approach emphasizes lateral regions of space where monaural spectral and head-shadow cues vary more strongly. Elevation response variability, *σ*, was quantified by calculating the standard deviation of the residuals around the best-fit line.

Furthermore, to quantify an overall measure of the response accuracy, we also used the azimuth mean absolute error (MAE, Equation 2) across trials, according to:


$$\mathrm{MAE}=\frac{1}{N}\sum_{n=1}^{N}|\alpha_{R}^{n}-\alpha_{T}^{n}|\;\;$$ (2)

With N the total number of trials and n each individual trial. MAE represents the average closeness of participants’ responses to the true target location.

The coefficient of determination (*r*^2^) was calculated to assess the strength of the target-response relationship, with higher *r*^2^ values indicating that a greater proportion of response variance is explained by the target location.

Azimuth outcomes were computed over the full azimuth range to obtain stable global estimates, whereas elevation was analyzed as a function of azimuth side (deaf/CI *vs.* hearing), to accurately quantify vertical performance within each 15° bin using elevation gain and response variability.

To measure the promptness of the localization trials, we recorded the reaction times (RT; in milliseconds) by calculating the difference between the onset of head movement and the onset of the target sound. We then computed the average of the reciprocal of each participant’s reaction time for each stimulus, to obtain s^−1^ values as a measure of promptness (1/RT), which have shown to follow a nearly Gaussian distribution (Carpenter et al., 2009; Carpenter and Williams, 1995).

A linear mixed-effects model was used to analyze differences in localization outcomes (gain, bias, *r*^2^, and MAE) across groups and stimulus intensity. The model included fixed effects for group, intensity, and their interaction, with subjects as a random effect to account for repeated measures. Pairwise comparisons between were conducted using estimated marginal means with Bonferroni-adjusted significance testing to control for multiple comparisons. Statistical significance was set at *p* < 0.05. All analyses were performed in SPSS Statistics (version 31, IBM Corp., Armonk, NY, USA). To evaluate whether the available sample size was sufficient for the mixed-effects design, we performed a *post hoc* Monte-Carlo simulation–based power analysis, defining power as the proportion of simulated datasets in which the relevant fixed effect (cohort, intensity, or cohort × intensity) reached significance at *p* < 0.05.

## 3 Results

Figure 1 shows a representative examples of the trial-by-trial localization behavior of a single participant from each of the listener groups in two dimensions (azimuth and elevation). Lines indicate the spatial trajectory of the head orienting response for each trial, where the localization response for sound sources presented from the right (red), center (grey), and left (blue) are represented. Typical localization behavior for a NH control is shown in Figure 1A where it can be observed the listener makes use of the entire front hemifield with correct right–left distinction. The listener with adult onset SSDa shows a typical response pattern with essentially all responses located to the better hearing ear, with a single orienting response towards the non-hearing side (Figure 1D). Notably, in the SSD-CI off, SSDa, and SSDc examples, sound sources presented on the deaf (left) side frequently result in head-orienting responses toward the NH (right) side, reflecting clear cross-hemifield localization errors. As expected, the SSD-CI listener demonstrates a significant improvement in azimuth performance when comparing CI on (Figure 1B) with CI off (Figure 1C). However, it’s noticeable that in either case responses are not distributed across elevation as for the control example. Furthermore, SSD-CI listener’s responses are clearly better distributed over the NH side (right) than on the CI side (left). Interestingly, the behavioral pattern for the SSDc listener demonstrates an ability to distinguish right and left targets not observed for the acquired (Figure 1E). Note that Figure 1 illustrates head-orienting trajectories but does not encode the accuracy of elevation responses relative to target location; elevation performance is therefore quantified separately using regression-based analyses (see section 3.2).

### 3.1 Horizontal sound localization

**Figure 2:**
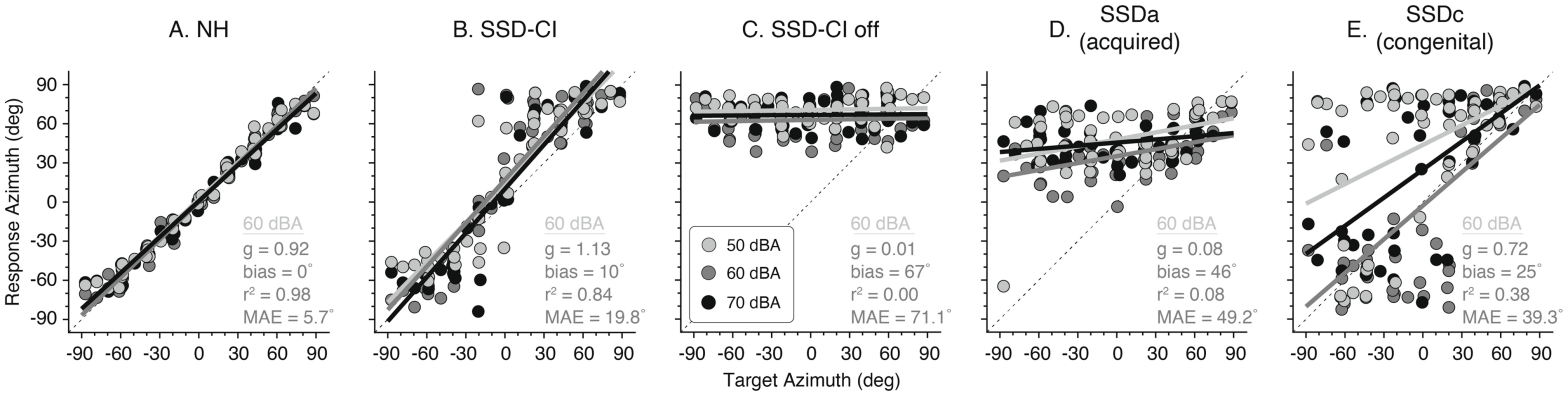
Associated azimuth target-response plots for the representative listener examples in Figure 1. **(A)** NH (normal hearing), **(B)** SSD-CI, **(C)** SSD-CI off, **(D)** SSDa (acquired), and **(E)** SSDc (congenital). Each color represents the presentation level. Azimuth gain, bias, r2, and MAE for 60 dBA are shown on each panel.

The associated stimulus–response relations in azimuth for the sample SSD listeners (Figure 1) are displayed in Figure 2. Linear regression analysis is displayed for the 0.5–20 kHz BB noise bursts of each SSD subject with stimulus intensity indicated by the grey scale. Performance in azimuth for the NH control demonstrates accurate and precise localization in azimuth (*g* = 0.92, bias = 0°, and *r*^2^ = 0.98), yielding a low MAE of 5.7° (Figure 2A). As observed in Figure 1D, the SSDa elicited a very strong bias toward the functional side (*b* = 46°) with low gain = 0.08 and *r*^2^ = 0.08 (Figure 2B). The SSD-CI listener demonstrates significant improvement in localization (Figure 2B) with the use of a CI with significantly higher gains, lower bias, and MAE improving from 71.1° (CI off, Figure 2C) to 19.8° (Figure 2C). Review of the unaided localization of the SSD-CI listener compared to the SSDa listener suggests slightly different strategies to localize sounds. Note that the dispersion of responses is not the same, with a more extreme bias observed for the unaided SSD-CI. Although there is a greater degree of error compared to the SSD-CI listener, the unaided SSDc listener behavior (Figure 2E) demonstrates an ability to lateralize the signal that is more consistent with the SSD-CI than the SSDa. This yields a much higher gain (*g* = 0.72), yet a better-ear bias persists (*b* = 25°) and a low *r*^2^ of 0.38 consistent with lateralization over localization of the signal.

**Figure 3:**
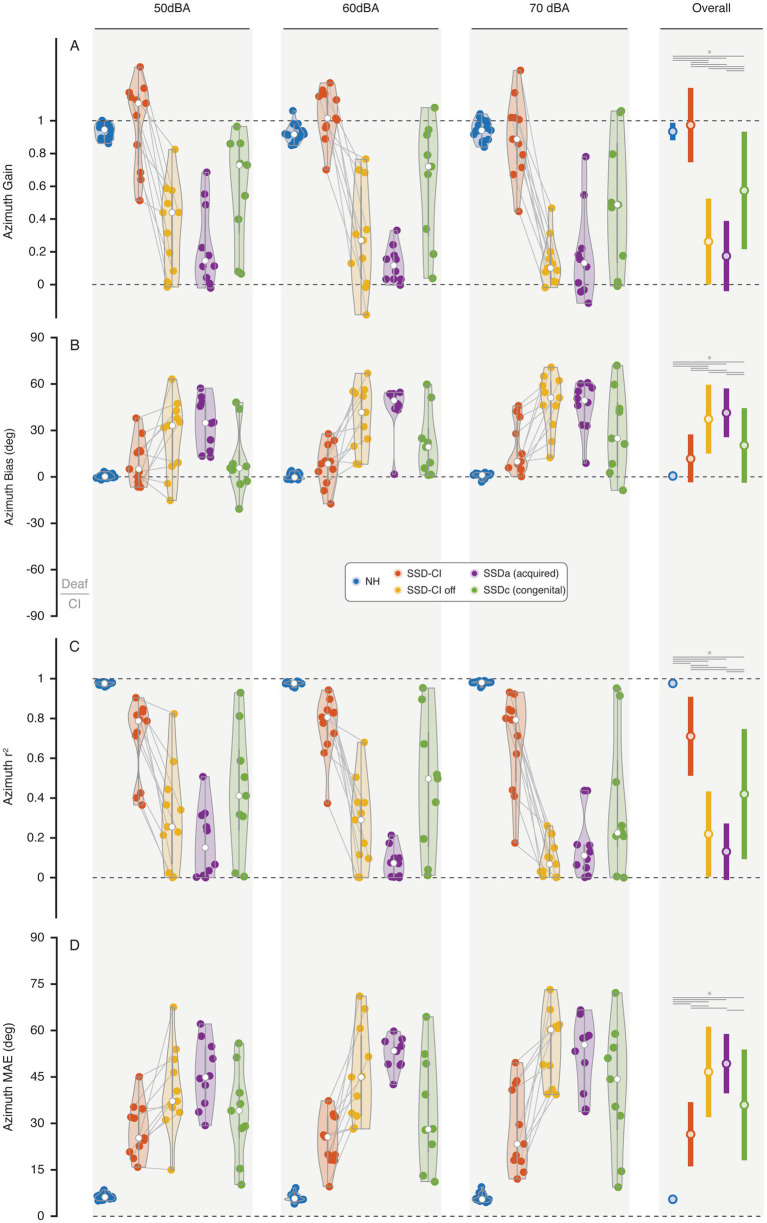
Azimuth localization outcomes for **(A)** gain, **(B)** bias, **(C)** *r*^2^, and **(D)** MAE for each presentation level, group, and overall means. *p < 0.05.

Figure 3 presents the azimuth regression results (gain, bias, *r*^2^ and MAE) for all subjects. Overall and as expected, NH showed accurate localization performance (Figure 3, blue) evidenced by an overall gain mean of 0.93 and mean *r*^2^ of 0.98, a mean bias of 0°, and a MAE of 6°. Analyses revealed a significant main effect of group [*F*(4, 55.16) = 108.62, *p* < 0.001] and stimulus intensity [*F*(2, 115.46) = 4.59, *p* = 0.012] on gain. The pairwise comparison showed a significant difference between SSD-CI listeners with the CI on and off (mean difference = 0.71, SE = 0.04, *p* < 0.001, 95% CI [0.598, 0.822]). Moreover, the SSD-CI off listening condition did not yield localization gain differences when compared with SSDa [mean difference = 0.088, SE = 0.081, *p* = 1, 95% CI (−0.151, 0.327)] but was lower than the congenital SSD listeners [mean difference = −0.311, SE = 0.085, *p* = 0.007, 95% CI (−0.563, −0.060)]. The SSDc group demonstrated higher localization gain (M = 0.57, SD = 0.35) compared to the SSDa group which exhibited the lowest gain across groups [(M = 0.17, SD = 0.16; mean difference = 0.399, SE = 0.085, *p* < 0.001, 95% CI [0.148, 0.651)]. Despite outperforming SSDa, the SSDc showed a significantly lower gain than the SSD-CI group (M = 0.97, SD = 0.19; mean difference = −0.399, SE = 0.085, *p* < 0.001, 95% CI [−0.650, −0.147]). Notably, there was no significant difference in localization gain between the SSD-CI group and NH listeners [(M = 0.93, SD = 0.05; mean difference = −0.040, SE = 0.074, *p* = 1, 95% CI [−0.259, 0.180)]. Note that azimuth gain values exceeding 1 reflect systematic response overshoot, rather than increased response variability, likely arising from over-weighting of available level-based or monaural cues for the SSD-CI listeners. Bonferroni-corrected post-hoc analysis revealed a significant difference between 50 and 70 dBA conditions (mean difference = 0.086, SE = 0.030, *p* = 0.014, 95% CI [0.014, 0.159]), with higher intensity (70 dBA) associated with lower gain and lower intensity (50 dBA) associated with higher gain. No stimulus intensity × group interaction was observed [*F*(8, 115.46) = 1.64, *p* = 0.121].

Significant group differences were observed in azimuth localization bias [*F*(4, 57.462) = 37.37, *p* < 0.001] alongside a main effect of stimulus intensity [*F*(2, 116.624) = 15.81, *p* < 0.001]. There was no significant stimulus intensity × group interaction [F(8, 115.46) = 1.640, *p* = 0.121]. NH exhibited minimal bias (M = 0.5°, SD = 1.6°). In the SSD-CI group there was a significant improvement from the CI on to CI off conditions [M = 37.3°, SD = 19.6°; mean difference = −25.4, SE = 2.826, *p* < 0.001, 95% CI (−33.487, −17.314)]. Although listeners maintained a bias towards the hearing side even with CI on (M = 11.9°, SD = 13.8°). No significant differences were found between SSD-CI off and SSDa [M = 41.4°, SD = 12.6°; mean difference = 4.078, SE = 5.482, *p* = 1, 95% CI (−12.074, 20.231)], or between SSDc (M = 20.3°, SD = 21.2°) and SSD-CI with CI on [mean difference = 8.401, SE = 5.778, *p* = 1, 95% CI (−8.626, 25.427)]. Stimulus intensity significantly affected bias, with pairwise comparisons showing differences between 50 dB *vs.* 60 dB [mean difference = −6.342, SE = 2.169, *p* = 0.012, 95% CI (−11.611, −1.073)], 50 dB *vs.* 70 dB [mean difference = −12.195, SE = 2.169, *p* < 0.001, 95% CI (−17.464, −6.926)], and 60 dB *vs.* 70 dB [mean difference = −5.853, SE = 2.169, *p* = 0.024, 95% CI (−11.122, −0.584)]. These results indicate an increase in response bias to the NH side as stimulus intensity increased.

The target-response relationship showed a significant main effect of group [*F*(4, 53.933) = 115.98, *p* < 0.001], intensity [*F*(2, 115.712) = 4.75, *p* = 0.010], and stimulus intensity × group interaction [*F*(8, 115.712) = 2.74, *p* = 0.008] for the analysis of goodness of linear fit (*r*^2^). NH achieved near perfect linearity (M = 0.98, SD = 0.01), followed by the SSD-CI with CI on (M = 0.71, SD = 0.19), though significantly more scattered around the linear fit than NH [(mean difference = 0.266, SE = 0.064, *p* = 0.002, 95% CI (0.076, 0.457)]. SSDc (M = 0.42, SD = 0.33) outperformed SSDa [M = 0.13, SD = 0.10; mean difference = −0.291, SE = 0.074, *p* = 0.003, 95% CI (−0.509, −0.072)], but was lower than NH [mean difference = −0.557, SE = 0.069, *p* < 0.001, 95% CI (−0.760, −0.355)]. No significant differences were observed between SSDc and SSD-CI off [M = 0.22, SD = 0.15; mean difference = 0.201, SE = 0.074, *p* = 0.093, 95% CI (−0.017, 0.420)] or between SSD-CI off and SSDa [mean difference = 0.089, SE = 0.070, *p* = 1.000, 95% CI (−0.119, 0.296)]. Intensity effects showed lower *r*^2^ at 70 dB compared to 50 dB [mean difference = −0.065, SE = 0.023, *p* = 0.016, 95% CI (−0.120, −0.009)] and 60 dB [mean difference = −0.056, SE = 0.023, *p* = 0.047, 95% CI (−0.111, −0.001)]. The interaction was driven by SSD-CI off, with significantly lower *r*^2^ at 70 dB compared to 50 dB [mean difference = −0.210, SE = 0.051, *p* < 0.001, 95% CI (0.085, 0.334)] and 60 dB [mean difference = −0.179, SE = 0.051, *p* = 0.002, 95% CI (0.054, 0.303)]. Other group × intensity pairwise comparisons were not significant (*p* ≥ 0.120).

Significant differences in MAE were observed across groups [*F*(4, 55.155) = 72.927, *p* < 0.001], with effects of intensity [*F*(2, 116.277) = 8.645, *p* < 0.001], and a stimulus intensity × group interaction [*F*(8, 116.277) = 2.611, *p* = 0.012]. NH listeners were the lowest MAE (M = 6.1°, SD = 1.1°) followed by the SSD-CI group with CI on (M = 26.9°, SD = 9.7°), though significantly higher than NH [mean difference = 20.84, SE = 3.64, *p* < 0.001, 95% CI (−31.59, −10.08)]. SSD-CI with CI on outperformed CI off [M = 46.9°, SD = 12.6°; mean difference = −20.00, SE = 1.78, *p* < 0.001, 95% CI (−25.09, −14.91)] and SSDa [M = 49.6°, SD = 5.7°; mean difference = −22.65, SE = 3.97, *p* < 0.001, 95% CI (−34.35, −10.94)]. SSDc (M = 36.4°, SD = 17.5°) showed no significant difference from SSD-CI with CI on [mean difference = −9.40, SE = 4.18, *p* = 0.295, 95% CI (−21.73, 2.94)], indicating better performance than SSDa. *Post hoc* comparisons revealed a significant increase in MAE at 70 dB (M = 26.3°) compared to both 50 dB [M = 30.7°; mean difference = 5.58°, SE = 1.36, *p* < 0.001, 95% CI [2.88, 8.28)] and 60 dB [M = 32.6°; mean difference = 3.69°, SE = 1.36, *p* = 0.001, 95% CI [0.99, 6.39)], whereas no significant difference was observed between 50 and 60 dB (*p* = 0.169). The stimulus intensity × group interaction was driven mainly by the SSD-CI off group, which showed an increase in MAE at 70 dB [M = 54.7°, SE = 3.32, 95% CI (48.1, 61.3)] compared to 50 dB [M = 40.7°, SE = 3.32, 95% CI (34.1, 47.3)] and 60 dB [M = 45.6°, SE = 3.32, 95% CI (39.0, 52.2)]. MAE changes across intensities were comparatively smaller in the other groups.

In summary, across all azimuth metrics (gain, bias, *r*^2^, and MAE), performance varied systematically by group and listening condition. NH listeners consistently showed near-ceiling performance. SSDa listeners exhibited the lowest gains, the largest biases toward the hearing ear, and the highest MAE. SSDc listeners showed intermediate performance, with higher gains and lower errors than SSDa but remaining clearly below NH. SSD-CI listeners demonstrated marked improvements with the implant on relative to CI off, characterized by higher gains, reduced bias, and lower MAE, although residual asymmetries and variability persisted compared with NH. Across groups, higher stimulus intensity was associated with lower gain, increased bias toward the hearing side, and larger MAE, with these effects being most pronounced in the SSD-CI off condition.

### 3.2 Vertical sound localization

**Figure 4:**
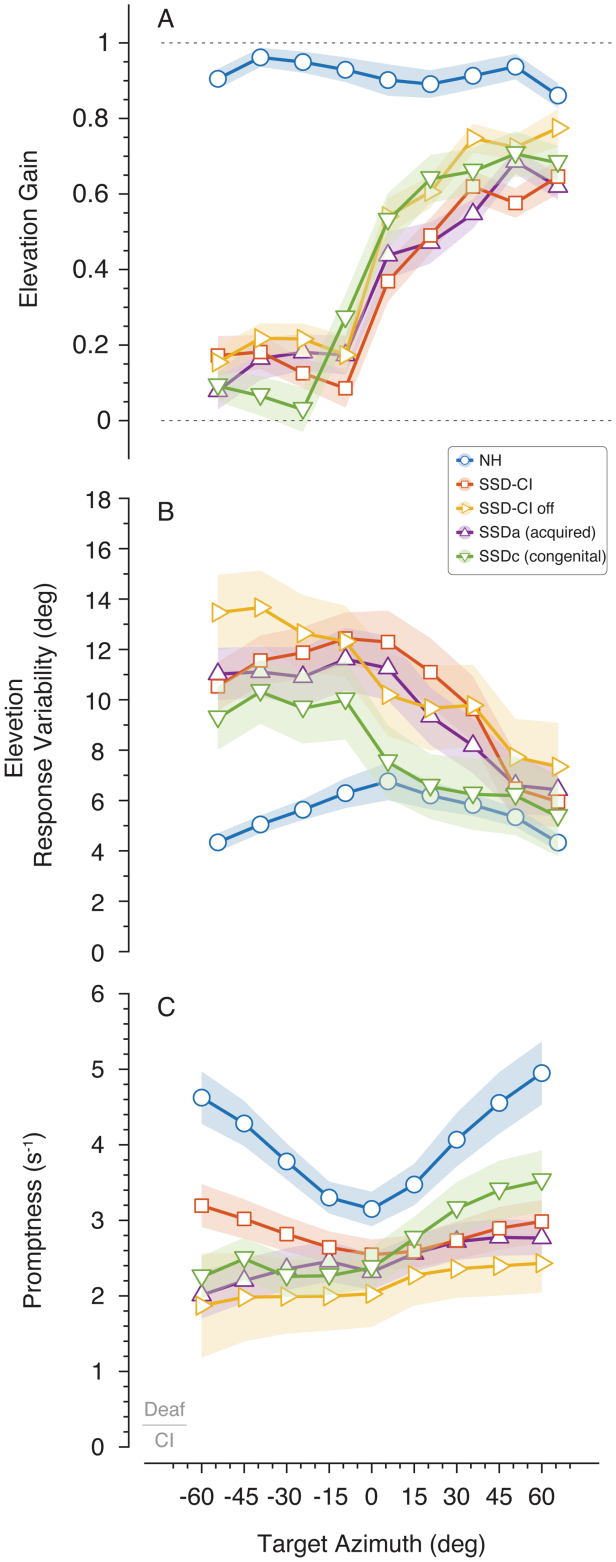
Elevation gain **(A)** and response variability **(B)** as a function of target azimuth. Mean promptness (inverse of reaction time) is also depicted as a function of target azimuth **(C)**, where greater values indicate increased promptness of the response. These metrics illustrate how vertical localization performance varies depending on whether the sound source is positioned to the deaf side (left) or NH side (right). The connected markers denote the binned mean across listeners with the shaded area indicating the standard error.

To examine the use of monaural spectral cues in SSD listeners, vertical sound localization performance was analyzed as a function of target azimuth location. Results are presented across different azimuthal positions to highlight how vertical localization varies depending on whether the sound source is positioned to the left or right of the listener. Figure 4 demonstrates this approach, with data normalized to represent the CI (i.e., deaf side) as the left ear (negative azimuthal angles, < −15°). NH listeners demonstrated accurate elevation localization across horizontal plane as evidenced by a mean gain of 0.92 (SD = 0.03) and a mean response variability of 5.5° (SD = 0.9°). Significant group differences were observed for elevation gain [*F*(4, 61.857) = 44.519, *p* < 0.001], side [*F*(1, 60.113) = 248.240, *p* < 0.001], and group × side interaction [*F*(4, 60.113) = 25.906, *p* < 0.001]. Response variability also revealed significant main effects of group [*F*(4, 49.3) = 4.67, *p* = 0.003], side [*F*(1, 63.7) = 66.52, *p* < 0.001], and group × side interaction [*F*(4, 63.7) = 7.69, *p* < 0.001]. Generally, SSD listeners demonstrated low gain on the deaf/CI side (M = 0.15, SD = 0.07) and high response variability (M = 11.5°, SD = 1.3°), improving on the hearing side (> 15°) to a mean gain of 0.64 (SD = 0.08) and response variability of 7.9° (SD = 1.7°). No significant differences were found among SSD groups on their deaf/CI side (*ɑ* < −15°) for gain (*p* > 0.656) or response variability (*p* > 0.161). On the hearing side (*ɑ* > 15°), the NH outperformed all SSD groups in gain (*p* > 0.037) but showed no significant difference in response variability (*p* > 0.542). Overall, elevation gain was uniformly low and variability high on the deaf/CI side across all SSD groups, whereas performance improved on the hearing side for all SSD listeners, though remaining below NH levels in gain.

### 3.3 Sound localization promptness

Response promptness to the auditory stimuli is presented for all participants in Figure 4C. Analyses revealed a significant main effect of group on localization promptness [*F*(4, 51.093) = 8.577, *p* < 0.001], side [*F*(1, 157.736) = 16.764, *p* < 0.001], with no group × side interaction [*F*(4, 157.736) = 0.836, *p* = 0.504]. In NH listeners, head movement speed during sound localization exhibited an expected U-shaped pattern across azimuth, known as eccentricity effect, with minimal movements for frontal sources and progressively faster movements toward lateral positions (Frens and Van Opstal, 1995; Gabriel et al., 2010; Goldring et al., 1996; Populin, 2008). NH listeners responded fastest (M = 4.02 s^−1^, SD = 0.63 s^−1^), while SSD-CI off was slowest (M = 2.15 s^−1^, SD = 0.22 s^−1^). On the deaf/CI side, SSD-CI with CI on showed significant improvement (M = 2.92 s^−1^, SD = 0.98 s^−1^) compared to CI off [M = 1.96 s^−1^, SD = 2.20 s^−1^; mean difference = 1.062, SE = 0.296, *p* = 0.006, 95% CI (0.202, 1.923)], with no significant difference from NH [mean difference = 1.218, SE = 0.504, *p* = 0.187, 95% CI (−0.251, 2.687)]. On the hearing side (*ɑ* > 15°), SSDc (M = 3.20 s^−1^, SD = 1.05 s^−1^) was comparable to NH [mean difference = 1.053, SE = 0.536, *p* = 0.542, 95% CI (−0.509, 2.616)]. Excluding SSD-CI with CI on, all other SSD groups (SSDa, SSDc, and SSD-CI off) exhibited slower responses on the deaf side (mean = 2.17 s^−1^, SD = 1.48) compared to the hearing side (M = 3.05 s^−1^, SD = 1.22 s^−1^). SSD-CI with CI on mirrored the eccentricity pattern observed in the NH group, showing minimal side differences in promptness (M left–right difference = 0.17 s^−1^, SD = 0.28 s^−1^). Overall, NH listeners demonstrated the fastest and most symmetric promptness across azimuth, whereas all SSD groups showed slower responses, particularly on the deaf side.

## 4 Discussion

The present study examined spatial hearing in individuals with SSDc, SSDa, and SSD-CI, focusing on azimuth and vertical localization performance, as well as response promptness. Our findings support the hypothesis that early cortical adaptation in SSDc enables stronger compensatory mechanisms compared to SSDa, as evidenced by superior azimuth localization gain (0.57 *vs.* 0.17), better goodness-of-fit (*r*^2^ = 0.70 *vs.* 0.41), and lower MAE (36° *vs.* 49.65°; see Figure 3). However, SSD-CI with CI on approached NH performance across metrics (gain = 0.97, *r*^2^ = 0.71, MAE = 27), highlighting the rehabilitative potential of CI despite partial binaural restoration. These results underscore the interplay between developmental plasticity, monaural cue reliance, and CI intervention in mitigating SSD deficits.

Figure 1 provides an informative visualization of individual localization behavior, displaying the actual head movement trajectories for each trial across azimuth and elevation. Unlike summary plots, this figure reveals the spatial strategies employed by each listener group in real time. SSDa responses are biased to the intact-ear hemifield, yet still span vertical space, consistent with monaural spectral cue use (Figure 1D). SSD-CI shows improved azimuth with CI on versus off (Figures 1B,C) but limited elevation spread. Notably, SSDc listener demonstrates a distinct and adaptive behavioral pattern. Even without intervention, the head movement trajectories are distributed across the auditory space, indicating accurate right–left discrimination. The SSDc listener, though less precise than NH, demonstrates robust lateralization, suggesting compensatory neural mechanisms unique to early-onset SSD. This finding highlights a unique compensatory adaptation in SSDc, setting it apart from SSDa and underscoring the heterogeneity of spatial hearing strategies in SSD listeners. Figure 2 provides individual regression analyses that quantify the accuracy and bias depicted in Figure 1, together illustrating both group × intensity patterns and individual adaptive differences in spatial hearing performance.

Critical periods in auditory development are characterized by heightened neuroplasticity, facilitating the calibration of binaural and monaural cues. In SSDc, early auditory deprivation induces cortical reorganization (Kral et al., 2013a), which enhances reliance on monaural spectral and head shadow cues. This neuroplastic adaptation enables select untreated individuals to achieve unbiased lateralization (Figure 1E) and near-normal localization metrics (e.g., high gain/*r*^2^ and low bias/ MAE; Figure 3) through reweighted cue processing and cross-modal integration (Slattery and Middlebrooks, 1994; Kumpik and King, 2019). In SSDa, where onset occurs after the critical period, the capacity for cortical remapping is restricted. As a result, individuals exhibit a strong bias toward the intact ear (Figure 1D and Figure 3B), reflecting the rigidity of established binaural processing pathways (Kumpik and King, 2019; Van Wanrooij and Opstal, 2004). Performance variability in SSDc likely reflects differences in auditory experience, neuroplasticity, and intact-ear high-frequency hearing.

In azimuth localization, NH listeners demonstrated near-perfect performance (gain = 0.93, *r*^2^ = 0.98, bias = 0.5°, MAE = 6°; see Figure 3), consistent with the integration of ITDs and ILDs (Blauert, 1996a; Blauert, 1996b). SSD groups without CI exhibited pronounced impairments, with biases toward the hearing side (SSDa: 33.3°, SSDc: 20°, SSD-CI off: 37.4°), reflecting reliance on monaural cues (Van Wanrooij and Opstal, 2004). The superior performance in SSDc over SSDa aligns with developmental plasticity theories, where early auditory deprivation prompts cortical reorganization to enhance monaural cue weighting (Keating and King, 2013; Kral et al., 2013a). This adaptation likely reduces functional handicap in SSDc, as predicted (Lieu, 2013), potentially explaining the moderate gains without CI intervention. However, these advantages may diminish in ecological settings with background noise and visual distractors, where SSDc and SSDa differences may be less pronounced, potentially reflecting increased task complexity and processing demands (Snapp and Ausili, 2020).

CI use dramatically improved SSD performance, reducing bias (12° *vs.* 37.4° in CI-off) and MAE (27° *vs.* 47°), with gains comparable to NH (*p* = 1.000). This supports prior evidence that CI partially restores binaural hearing, primarily through ILDs, enhancing localization (Dorman et al., 2015). Improvements in CI-on vs. CI-off conditions corroborate studies showing marked localization benefits with CI use (Távora-Vieira et al., 2015; Litovsky et al., 2019; Sullivan et al., 2020; Daher et al., 2023; Oh et al., 2023; Van Heteren et al., 2024). However, CI does not completely restore binaural integration, as evidenced by slower reaction times (303 ms *vs.* NH 236 ms on deaf side), lower *r*^2^ (0.71 *vs.* 0.98, *p* = 0.002), persistent bias (12°), elevated MAE (27° *vs.* 6°), and increased response variability compared to NH. These limitations reflect CI constraints in spectral resolution and ITD processing (Francart et al., 2018), leading to incomplete binaural restoration (Snapp and Ausili, 2020). Slower promptness in SSD groups may reflect uncertainty in cue extraction or increased processing demands, particularly in SSD-CI, where processing acoustic and contralateral electrical inputs may introduce interference in complex settings (Pichora-Fuller et al., 2016; Ausili and Snapp, 2023). The observed stimulus intensity effects with reduced *r*^2^ and increased bias at higher intensities (e.g., 70 dB *vs.* 50 dB: *r*^2^ difference = −0.065, *p* = 0.016), suggest intensity-dependent cue saliency, particularly in SSD-CI off, where the interaction was driven (*p* < 0.001). This intensity dependence behavior is consistent with monaural localization strategies. In the absence of binaural cues, listeners rely predominantly on head-shadow-derived level cues and monaural spectral information, which change systematically with sound level but are inherently ambiguous for spatial decoding. At higher presentation intensities, these monaural cues can saturate or shift the internal mapping between acoustic level and perceived azimuth, leading to degraded regression fits and larger localization errors in SSD listeners (Van Wanrooij and Opstal, 2004).

Vertical localization, reliant on monaural spectral cues, revealed side-specific deficits in SSD groups, with low gain (0.15) and high response variability (11.5°) on the deaf side, with performance improving on the hearing side (gain = 0.64, variability = 7.9°; Figure 4). No differences among SSD groups on the deaf side (*p* > 0.161) indicate uniform limitations in spectral cue access without binaural support, consistent with pinna filtering dominance (Blauert, 1996a; Blauert, 1996b). NH superiority on the hearing side (*p* < 0.037 for gain) underscores intact binaural integration. The hearing-side improvement in SSD aligns with the facilitation of spectral cues (Van Wanrooij and Opstal, 2004; Ausili et al., 2019; Ausili and Snapp, 2023). As anticipated, CI activation did not significantly enhance vertical localization performance, with SSD-CI off conditions mirroring other SSD groups, indicating that CI primarily facilitates horizontal localization through ILDs rather than improving monaural spectral cue processing (Dorman et al., 2015).

Response promptness provided insight into adaptive efficiency (Figure 4). NH responded fastest (4.02 s^−1^, 249 ms), while SSD-CI off was slowest (2.15 s^−1^, 465 ms). In NH individuals, robust binaural timing and level differences facilitate rapid head-orienting responses that increase as targets move off midline due to enhanced binaural disparities, yielding the u-shaped eccentricity profile (Figure 4; Ausili et al., 2019). SSD disrupts this binaural computation, resulting in prolonged latencies and flattened patterns, particularly for more lateral targets. CI activation improved deaf-side promptness (2.92 s^−1^, 343 ms; *p* = 0.006 *vs.* CI-off), approaching NH levels (*p* = 0.187), indicating that bilateral facilitation reduces processing effort. SSDc was comparable to NH on the hearing side (3.20 s^−1^, 313 ms; *p* = 0.542), reflecting compensatory monaural adaptations (Keating and King, 2013; Tillein et al., 2016). Congenital onset of deafness leads to neuroplastic reorganization in the auditory brainstem and cortex, optimizing monaural processing for intact-side stimuli (Keating and King, 2013; Tillein et al., 2016). Conversely, SSDa demonstrated persistent slowed responses on the NH side, potentially reflecting less effective compensatory reliance on monaural spectral cues from the intact ear compared to SSDc. Slower response rates on the impaired side in non-CI SSD (deaf: 2.17 s^−1^, 460 ms; vs. hearing: 3.05 s^−1^, 328 ms) were minimized in SSD-CI (difference = 0.17 s^−1^), mirroring NH eccentricity patterns (Ausili et al., 2019; Ausili and Snapp, 2023). This suggests CI not only improves accuracy but also promptness, potentially via cortical remapping (Kral et al., 2013a).

The findings suggest early compensation in SSDc mitigates deficits, but may also constrain the restorative potential of CI, as SSDc MAE (overall mean = 36°) was comparable to SSD-CI (overall mean = 27°, *p* = 0.295), unlike sharper benefits observed in SSDa. This supports etiology-dependent expectations for CI benefit, with greater room for improvement in post-developmental cases (Lieu, 2013; Litovsky et al., 2019). Notably, the lack of robust evidence on spatial hearing abilities in congenitally deafened listeners, limits our understanding of their performance in real-world scenarios (Snapp and Ausili, 2020). SSD-CI listeners may face additional challenges processing combined acoustic and electrical inputs in such environments, necessitating ecologically valid testing to capture true performance differences.

Importantly, the localization metrics examined capture complementary aspects of spatial hearing behavior that are relevant for clinical interpretation. Rather than reflecting a single dimension of performance, these measures together help disentangle accuracy, consistency, and systematic spatial biases, providing a more comprehensive characterization of localization strategies in SSD. Taken together, the combined assessment of azimuth accuracy, bias, consistency, elevation mapping, and response promptness provides a multidimensional profile of spatial-hearing function that may help identify individuals with SSD who have limited versus substantial potential benefit from CI.

## Data Availability

The raw data supporting the conclusions of this article will be made available by the authors, without undue reservation.
